# The effect of polyglycolic acid sheet in preventing postoperative recurrent pneumothorax: a prospective cohort study

**DOI:** 10.1186/s13019-023-02111-w

**Published:** 2023-01-10

**Authors:** Takafumi Kabuto, Mitsugu Omasa, Shunichi Nagata, Kosuke Tokushige, Taishi Adachi, Takao Nakanishi, Hideki Motoyama

**Affiliations:** 1 Department of Thoracic Surgery, Kobe City Nishi-Kobe Medical Center, 5-7-1, Kojidai, Nishi-Ku, Kobe, Hyogo 651-2273 Japan; 2 Department of Thoracic Surgery, Nagara Medical Center, 1300-7 Nagara, Gifu, 502-8558 Japan; 3 Department of Respiratory Medicine, Kobe City Nishi-Kobe Medical Center, 5-7-1, Kojidai, Nishi-Ku, Kobe, Hyogo 651-2273 Japan

**Keywords:** Pneumothorax, VATS, Polyglycolic acid sheet, Oxidized regenerated cellulose sheet, Recurrence

## Abstract

**Background:**

Primary spontaneous pneumothorax occasionally relapses, even after bullectomy; therefore, coverage of the bullectomy staple line for pleural reinforcement is common in Japan. However, the appropriate covering materials have not yet been determined.

**Methods:**

This was a longitudinal prospective cohort study. Data were available for patients aged < 40 years with primary spontaneous pneumothorax who underwent their first thoracoscopic bullectomy between July 2015 and June 2021. We used oxidized regenerated cellulose (ORC) sheets from July 2015 to June 2018, and polyglycolic acid (PGA) sheets from July 2018 to June 2021. The postoperative recurrence-free survival rate was evaluated. The characteristics of the recurrent cases (radiographic, intraoperative, and pathological findings) were also evaluated. The extent of pleural adhesions was classified into the following three groups: none, medium, or extensive.

**Results:**

A total of 187 patients were included in the study. There were 92 and 95 participants in the ORC and PGA sheet groups, respectively. The postoperative recurrence-free survival rates were significantly higher in the PGA sheet group than in the ORC sheet group (ORC group vs. PGA group, 82.9% vs. 95.4%, *p* = 0.031). In recurrent cases, there was a significant difference in terms of pleural adhesion (0.0% [12 of 12, none] vs. 100.0% [four of four, extensive], *p* < 0.001).

**Conclusions:**

Compared with ORC sheets, PGA sheets are an effective material for preventing early recurrence of primary spontaneous pneumothorax. Strong local pleural adhesions potentially contribute to decreasing recurrence.

## Background

Primary spontaneous pneumothorax (PSP) recurs more frequently in young patients than in older patients when video-assisted thoracoscopic surgery (VATS) bullectomy is performed [1]. According European Respiratory Society’s guideline, chemical or mechanical pleurodesis during surgery might be a general technique; however, these methods have some complications, for example, too severe pleural adhesions or parietal bleeding [2–4]. In Japan, reinforcement of the staple line by sheet coverage is commonly performed during surgery [5]. We previously reported that oxidized regenerated cellulose (ORC) sheets thicken the visceral pleura but cannot prevent recurrence of PSP [6]. Another covering material on the staple line may be effective in preventing recurrence, and several studies have reported on appropriate materials [7–10]. Our research on covering materials and PSPs has also continued. A polyglycolic acid (PGA) sheet is an absorbable and reinforcement material that is hydrolyzed and employed at the time of postoperative air leakage repair [11, 12]. In the current prospective cohort study, we evaluated the efficiency of PGA sheets for the prevention of early recurrence after bullectomy in young patients with PSP compared to that of ORC sheets.

## Methods

### Patient and group classification

This is a prospective cohort study using data from Kobe City Nishi-Kobe Medical Center from July 2015 to June 2021. During this period, we performed VATS bullectomy as a primary surgery for PSP in patients aged < 40 years at our institution. The patients were diagnosed as PSP clinically and radiographically, and patients with some pulmonary diseases, such as pulmonary infections, emphysema, interstitial pneumonia, malignant diseases, catamenial pneumothorax, lymphangioleiomyomatosis, Birt–Hogg–Dubé syndrome, or Marfan’s syndrome, were excluded. The following were the surgical-indication criteria for PSP: persistent air leaks (> 48 h), recurrent pneumothorax, and a bulla detected on chest computed tomography.

**Fig. 1:**
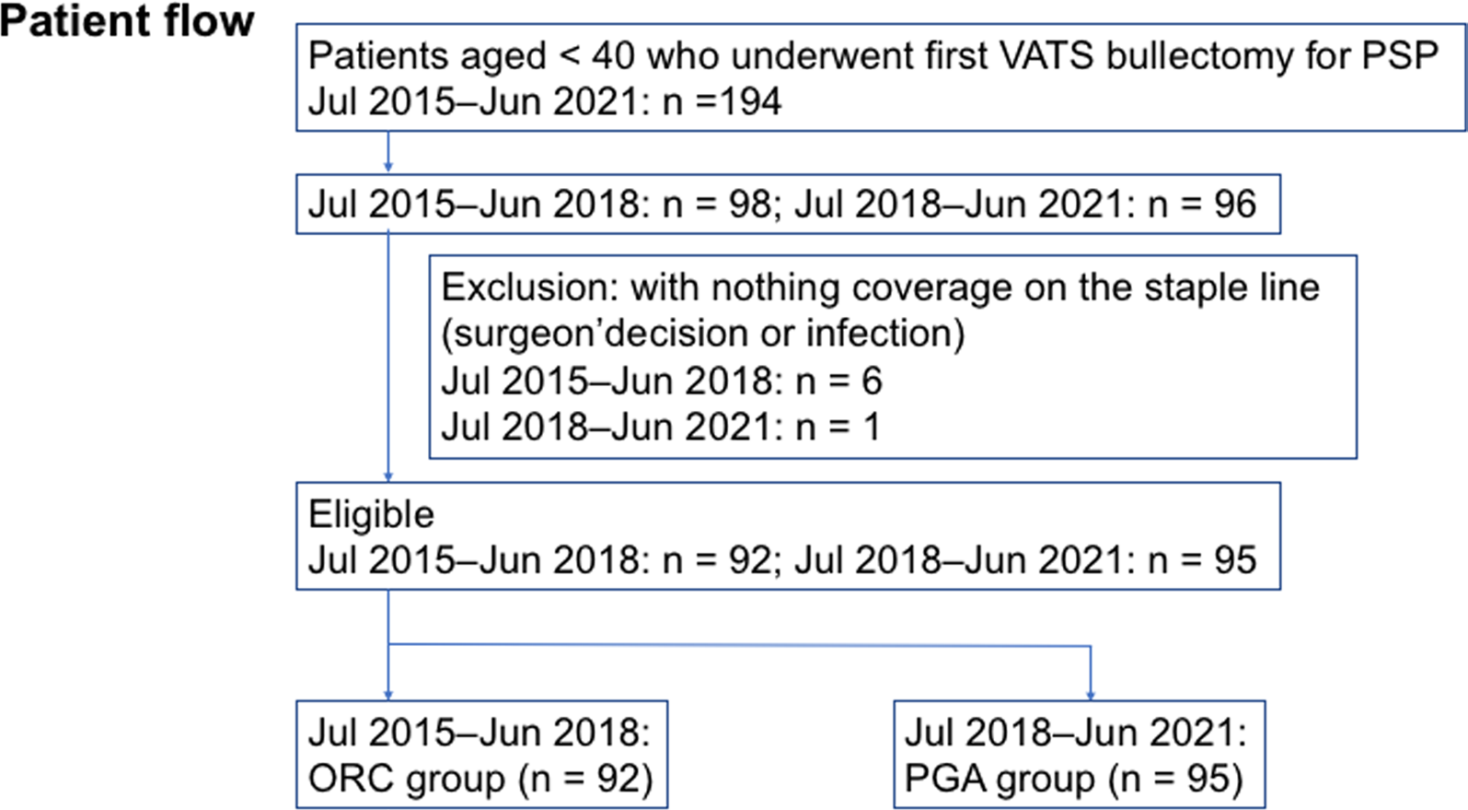
Patient flowchart. VATS, video-assisted thoracic surgery; PSP, primary spontaneous pneumothorax; ORC, oxidized regenerated cellulose; PGA, polyglycolic acid

We prospectively used an ORC sheet (SURGICEL^®^ Original Absorbable Hemostat, 2 × 3 in; Johnson & Johnson K. K., Tokyo, Japan) from July 2015 to June 2018, and a PGA sheet (NEOVEIL^®^, Gunze Ltd., Kyoto, Japan) from July 2018 to June 2021. Bulla resection and sheet coverage were performed on the staple line in both groups in a similar fashion. We excluded patients whose staple lines were covered with nothing or other materials for any reason (surgeon’s decision or infection) (shown in Fig. 1). A minimal amount of autologous blood (approximately 5–10 mL) was added for sheet fixation, whenever required.

### Follow-up

After the initial surgery, we instructed all patients to visit our institution when they experienced symptoms similar to those of pneumothorax, such as chest pain or dyspnea. We defined postoperative recurrence as ipsilateral pneumothorax more than 30 days after the surgery date detected in the medical chart or radiological examinations at our institution.

### Variables and outcomes

Clinical information included patient profile data, radiological images, intraoperative findings, postoperative recurrence, and postoperative complications, according to the Clavien-Dindo classification.

The primary outcome was the recurrence-free pneumothorax rate after bullectomy. The recurrence-free time spanned from the surgery date to June 30, 2018, in the ORC group and June 30, 2021, in the PGA group.

Recurrence characteristics (pleural adhesion, bulla regeneration, location of the regenerated bulla, and treatment for recurrent pneumothorax) observed in the radiological or intraoperative findings were also evaluated using medical charts, thoracic radiological examinations, intraoperative movies, and pathological findings. The extent of pleural adhesion was classified into the following three groups: nothing or funicular adhesions as “none,” approximately 0–3 cm as “medium,” and approximately > 3 cm as “extensive” in radiological or intraoperative findings.

### Statistical analysis

Differences between groups with respect to normally distributed continuous variables were assessed using the t-test. Categorical variables were analyzed using Fisher’s exact test or the Chi-squared test, as appropriate. The Kaplan–Meier method and log-rank tests were used to analyze recurrence-free survival curves. All *p*-values were two-sided, and statistical significance was set at *p* < 0.05. All statistical analyses were performed using JMP (version16; SAS Institute Inc., Cary, NC, USA).

## Results

### Patient characteristics

During this study period, 194 patients underwent their first VATS bullectomy, and seven were excluded due to lack of use of the specific sheet in each period (six in the period from July 2015 to June 2018 due to the surgeon’s decision and one in the period from July 2018 to June 2021 due to contaminated pleural effusion). In this study, 187 patients (92 in the ORC group and 95 in the PGA group) were included (shown in Fig. 1).

Table 1 summarizes the patient characteristics. There were no significant differences between the two groups in terms of age (years, 21.0 [19.7–22.3] vs. 22.5 [21.2–23.8], *p* = 0.12), sex (male, 84 [91.3%] vs. 86 [90.5%], *p* = 0.85), mean observation time (days, 622.0 [558.0–685.9] vs. 615.7 [552.7–678.7], *p* = 0.89), and postoperative complications with Clavien-Dindo classification grade > grade II (one, postoperative bleeding [1.1%] vs. one, empyema [1.1%], *p* = 1). There were no cases of mortality in either group.

**Table 1:** Characteristics of two groups

	ORC group (n = 92)	PGA group (n = 95)	*P* value
Age, mean (years)	21.0 (19.7–22.3)	22.5 (21.2–23.8)	0.12
Sex (male)	84 (91.3%)	86 (90.5%)	0.85
Side (left)	50 (54.4%)	53 (55.8%)	0.84
Observation time, mean (days)	621.9 (558.0–685.9)	615.7 (552.7–678.7)	0.89
Postoperative complication (CD classification grade > grade II)	1 (1.1%)	1 (1.1%)	1

CD classification, Clavien-Dindo classification; ORC, oxidized regenerated cellulose; PGA, polyglycolic acid

### Postoperative recurrence of pneumothorax

**Fig. 2:**
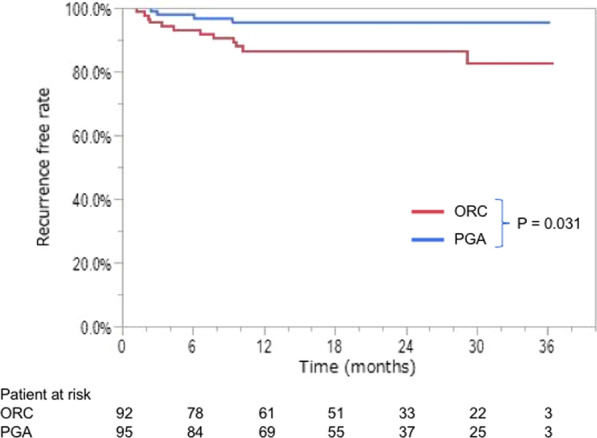
Recurrence-free survival curves. ORC, oxidized regenerated cellulose; PGA, polyglycolic acid

Figure 2 shows the recurrence-free survival curves. The 3-year postoperative recurrence-free rates were 82.9% and 95.4% in the ORC and PGA groups, respectively (*p* = 0.031).

### Intraoperative and radiographical findings of postoperative recurrent cases

**Fig. 3:**
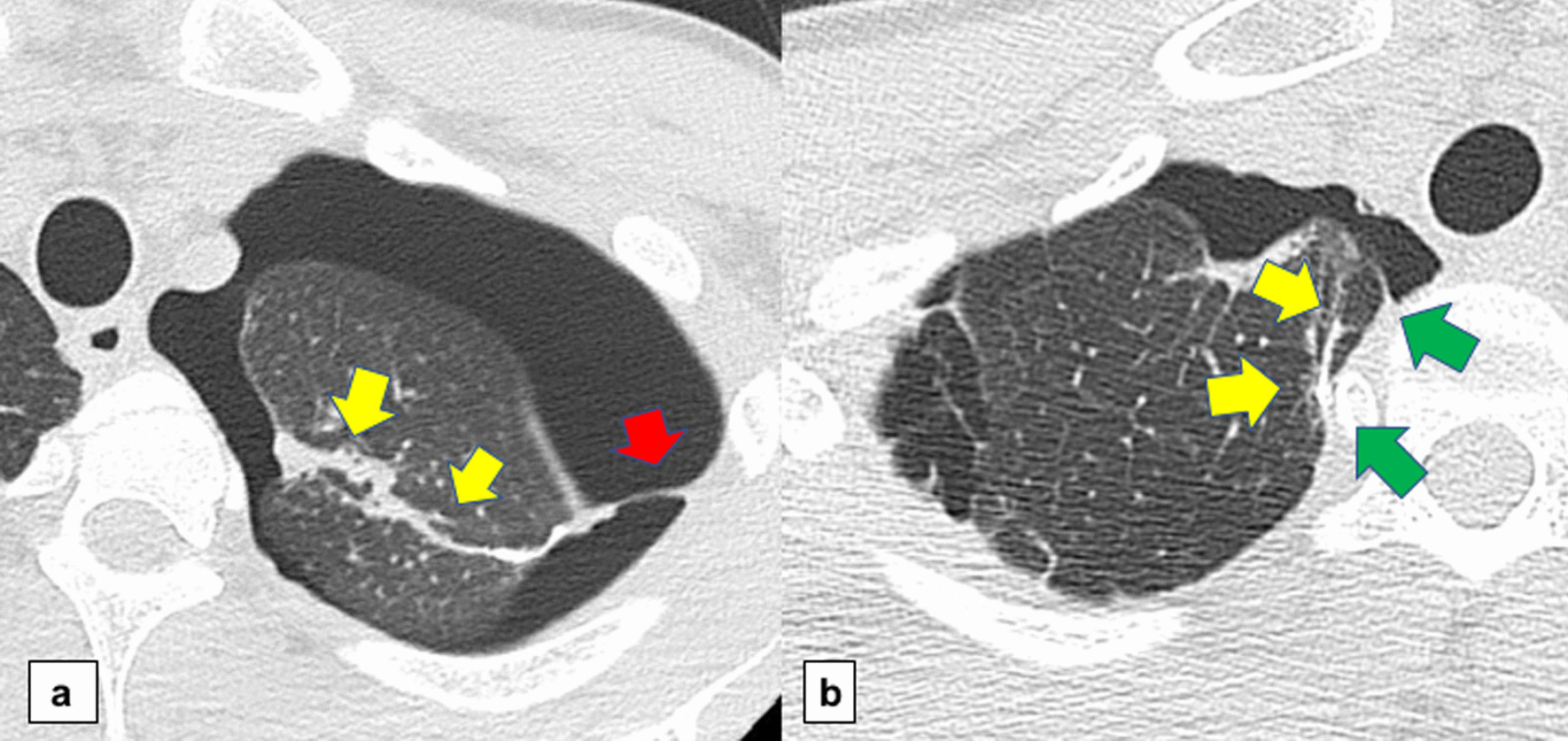
Computed tomography images at the time of recurrence. Yellow arrows indicate the staple line in each image. **a**. ORC coverage: only funicular adhesion (red arrow) was observed. **b**. PGA coverage: covered lung was tightly adhered to the chest wall, and green arrows indicate the thickened pleura. ORC, oxidized regenerated cellulose; PGA, polyglycolic acid

The data of patients with postoperative recurrent pneumothorax are provided in Table 2. In the ORC group, none of the patients had medium or extensive adhesion on radiological images (only two patients had funicular adhesion). In contrast, all four patients with recurrence in the PGA group exhibited extensive pleural adhesions at the coverage area (0.0% vs. 100.0%, *p* < 0.001) (shown in Fig. 3). There was no significant difference between the two groups in terms of recurrent pneumothorax treatment (observation/intervention, 9/3 vs. 1/3, *p* = 0.12).

**Table 2:** Summary of recurrent cases

	ORC group (n = 92)	PGA group (n = 95)	*P* value
Recurrence	12	4	0.031*
Recurrence free time (days)	220.2 (91.8–348.7)	156.5 (-66.0–379.0)	0.60
Treatment			
Observation	9 (75.0%)	1 (25.0%)	0.12
Intervention	3 (25.0%)	3 (75.0%)	
Pleurodesis	1	1	
Reoperation	2	2	
Pleural adhesion (none/ medium/ extensive)	12 (100.0%) / 0/ 0	0/ 0/ 4 (100.0%)	< 0.001
Bulla regeneration	5	1	
Regenerated bulla and the staple line			
Close	3	0	
Unrelated	3	1	

ORC, oxidized regenerated cellulose; PGA, polyglycolic acid

*log-rank test

In the ORC group, the regenerated bulla was close to the staple line in two patients and unrelated in three patients. In the PGA group, bulla neogenesis unrelated to the staple line was detected in one patient.

**Fig. 4:**
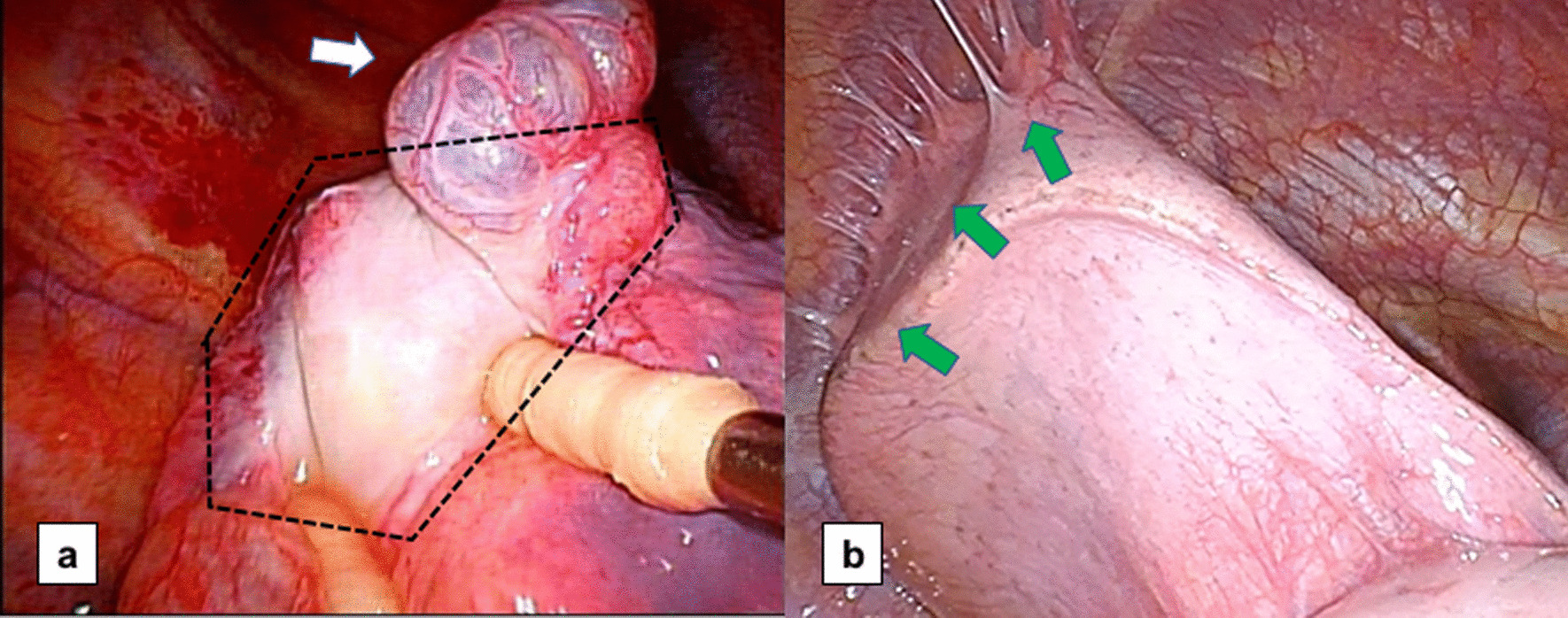
Intraoperative findings during reoperation. **a**. ORC: the covered visceral pleura circled by the dotted line was thickened. Newly generated bullae were observed (white arrow). **b**. PGA: lung covered by the PGA sheet tightly adhered to the parietal pleura (green arrows). ORC, oxidized regenerated cellulose; PGA, polyglycolic acid

**Fig. 5:**
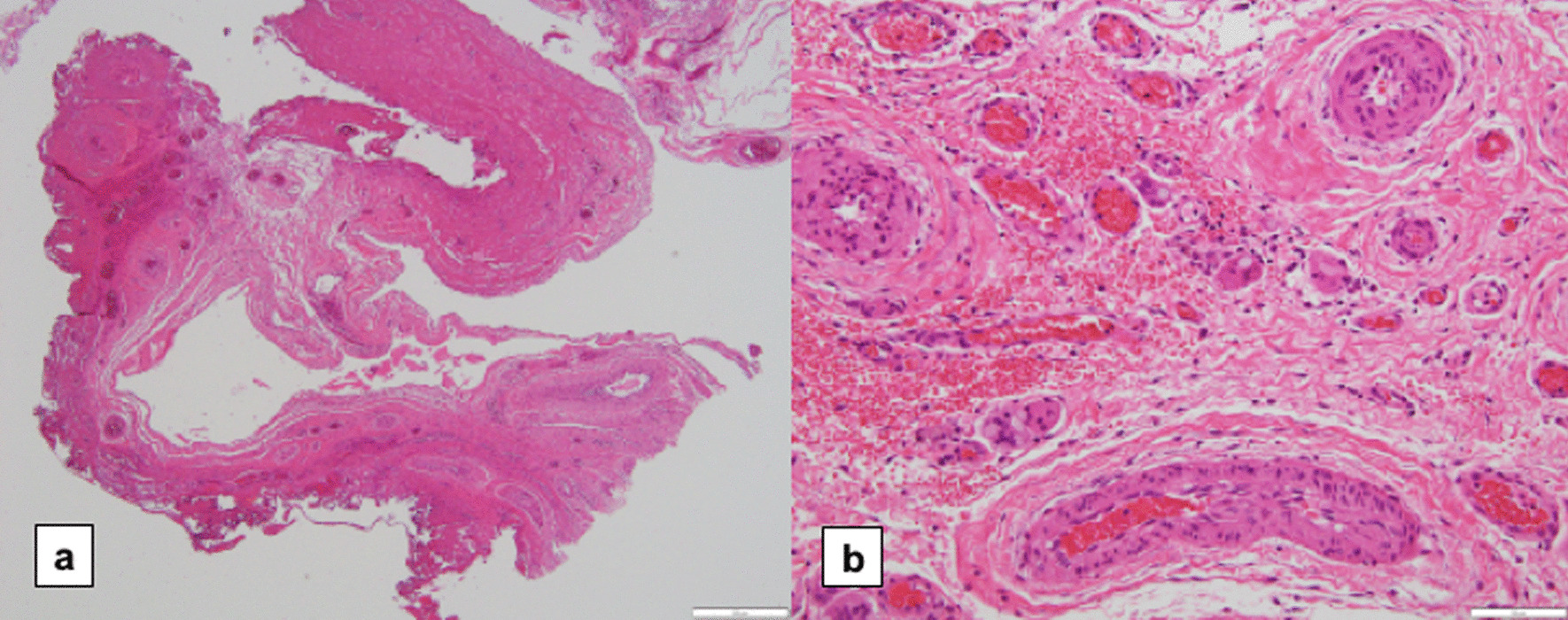
Pathological specimen of the resected pleura that was covered by a polyglycolic acid sheet. **a**. hematoxylin–eosin staining, × 4: inflammatory thickened visceral pleura. **b**. hematoxylin–eosin staining, × 20: fibrosis and angiogenesis were found

At the time of reoperation, the pleural adhesion caused by the PGA sheet was extensive and tight (shown in Fig. 4). The pathological specimen of the area covered by the PGA sheet revealed thickened visceral pleura with high fibrosis and angiogenesis. Moreover, a foreign body reaction and inflammatory cell infiltration (lymphocytes, neutrophils, and eosinophils) were observed (shown in Fig. 5).

## Discussion

Postoperative recurrent pneumothorax after bullectomy remains a clinical challenge, especially in young patients because they have a higher recurrence rate than in older patients [1]. The recurrence rate of bullectomy with no coverage in young patients is reported to be as high as 10.9–19.5% [6, 7, 9]. The etiology of postoperative recurrence of PSP is speculated to be rupture of the overlooked or regenerated bulla, which is formed at the staple line of the bullectomy. Therefore, we continued our research on appropriate covering materials for bullectomy staple lines; however, we could not find evidence of postoperative-recurrence suppression by ORC sheets. This finding was supported by our previous study [6].

This study demonstrated the superior effectiveness of the PGA sheet to that of the ORC sheet. The PGA sheet significantly decreased the recurrence rate of pneumothorax after bullectomy compared with the ORC sheet. PGA sheets have been reported to exhibit a limited effect on bulla regeneration [13]; nevertheless, recurrent cases in this study implied that the sheet was possibly more efficient in inducing pleural adhesion than the ORC sheet. We speculated that the pleural adhesion in the coverage area leads to reduced postoperative recurrence.

The PGA sheet is an absorbable reinforcement material for the pleura [11]. The sheet was pathologically proven to cause inflammatory cell infiltration with pleural fibrosis in this recurrent case. This indicated that the PGA sheet triggers inflammation in the visceral pleura, leading to the formation of strong and extensive pleural adhesions. Its use has not demonstrated an increase in surgical site infection [14]. PGA sheets on bulla have been reported to induce the formation of granulation tissue and reinforce the bulla wall 1 month after surgery in the case of secondary spontaneous pneumothorax [15]. In contrast, the ORC sheet just thickens the visceral pleura by fibrous hyperplasia and does not contribute to creating pleural adhesion or reducing postoperative recurrence as we previously reported [6].

Several previous reports, including the most recent meta-analysis, have demonstrated the effectiveness of PGA sheets in preventing postoperative recurrence compared with other methods [7–10]. However, these studies were retrospective, and none could use their data to establish the reason PGA sheets potentially suppress recurrence. Our study was conducted using a prospective cohort approach and provided precise radiological, intraoperative, and pathological information on recurrent cases. The methods of bulla resection, sheet coverage, and follow-up were the same in the prospective cohorts, and we included patients whose characteristics were similar in the fixed local region. Therefore, we were able to assess both groups equally.

We also reported that the ORC sheet only has a limited effect on suppressing the regenerating bulla on the staple line [6]. Thus, we expected a significant effect from a better covering material. The resected visceral pleura at the time of bullectomy is weakened by tensional force, which is responsible for bulla regeneration, even under the covering sheet [16]. Although the number of recurrent cases is limited, this study implied that PGA sheets have an equivalent suppressive effect on bulla regeneration to ORC sheets. PGA sheets reportedly do not prevent bulla regeneration, which is a risk factor for recurrence, even though it decreases the postoperative recurrence rate [16, 17]. Therefore, PGA sheets are believed to prevent postoperative recurrence by preventing bullae from rupturing.

Pleural adhesion may have an important influence on preventing air leakage from the staple line, and it is produced by various techniques. In European countries, thoracoscopic chemical pleurodesis using talc poudrage or mechanical pleurodesis (pleural abrasion and pleurectomy) is a common method because some studies have demonstrated its workability [2]. The recurrence rate may be low after VATS with talc poudrage; however, reoperation is difficult due to tight pleural adhesions at the time of reoperation [17]. Extensive and tight pleural adhesions are potential obstacles at the time of the second thoracic surgery, such as otitis lung injury, bleeding, and surgical time. Moreover, good pleurodesis reagents, such as talc, are not covered for PSP by the health insurance system in Japan. Therefore, pleurodesis for PSP often fails [1]. Mechanical pleurodesis is also known as a procedure to induce pleural adhesion, but its complications include postoperative chest pain, intra- and postoperative bleeding [4]. The severe adhesions can complicate the future surgery. Additionally, it was proven that mechanical pleurodesis does not have the significant effect to prevent recurrent pneumothorax. Although the local adhesion created by the PGA sheet may be disadvantageous and challenging, reoperation after PGA-sheet coverage seems easier than that of pleurodesis because of the limited adhesion area. Therefore, we consider that pleural adhesion is necessary; however, the adhesion area should be maintained at a minimum. A future clinical investigation comparing PGA sheet coverage with intraoperative pleurodesis is required.

### Limitations

This study is limited by its single-center design. As in our previous study, most of the postoperative recurrence occurred within 2 years [6]. The observation period was limited to 3 years; nonetheless, it was a sufficient period for evaluating the presence of postoperative recurrence.

In previous studies on the recurrence of postoperative pneumothorax, follow-up by telephone or routine checkups might have been common. However, these methods fail to reveal the true recurrence rate because they cannot detect minor or asymptomatic recurrence. In this study, almost all patients visited our institution when they experienced any symptoms, such as dyspnea or chest pain, because our institution is the only medical center that can manage pneumothorax in the region. Therefore, follow-up through emergent visits, together with the observation of certain symptoms, was sufficient to compare treatment outcome in this study. This method was approved in our previous study [6].

## Conclusion

A PGA sheet covering the staple line after bullectomy is more effective than an ORC sheet for preventing the early recurrence of PSP in young patients. Both sheets demonstrated a limited effect in suppressing bulla regeneration, although only the PGA sheet caused tight pleural adhesion at the coverage area. Local strong pleural adhesions potentially contribute to the decreased recurrence of PSP.

## Data Availability

The datasets used and/or analyzed during the current study are available from the corresponding author on reasonable request.

## Abbreviations

PSP: Primary spontaneous pneumothorax
VATS: Video-assisted thoracoscopic surgery
ORC: Oxidized regenerated cellulose
PGA: Polyglycolic acid
